# Genetic variation in and relationships among faecal worm eggs recorded in different seasons of the year at the Tygerhoek farm in South Africa

**DOI:** 10.4102/jsava.v88i0.1484

**Published:** 2017-07-07

**Authors:** Ziyanda Mpetile, Kennedy Dzama, Schalk W.P. Cloete

**Affiliations:** 1 Department of Animal Sciences, Stellenbosch University, South Africa; 2 Elsenburg, Western Cape Department of Agriculture, South Africa

## Abstract

Gastrointestinal nematodes result in severe economic and production losses to the sheep industry. An increase in resistance of the nematodes to chemicals used for control, as well as a demand of consumers for meat products free from chemicals, has fostered research on alternative control strategies. Breeding for resistance to nematodes offers an alternative to control parasitism but its effectiveness depends on genetic variation in faecal worm egg count (FWEC), an indirect measure of parasite resistance. A historic dataset of FWEC from four Merino lines subjected to natural parasite challenge was used to estimate genetic parameters for FWEC in three seasons (autumn, winter and spring) using a repeated records animal model, followed by a three-trait animal model analysis treating FWEC in different seasons as separate traits. The effects of selection line, birth year, sex, the sex x birth year interaction, season and the season x year interaction were significant when using 4994 records recorded from 1997 to 2000 (*p* < 0.001). The heritability of log-transformed FWEC amounted to 0.09 ± 0.02, with no contribution from the animal permanent environmental variance to the between animal variation across seasons. Three-trait heritability estimates for log-transformed FWEC amounted to 0.07 ± 0.05 in autumn, 0.13 ± 0.05 in winter and 0.19 ± 0.05 in spring. These results suggest sufficient genetic variation in FWEC to support selection for lower log-transformed FWEC. However, the best time to record data for selection is after the break of the season in winter and in spring, when sheep are stimulated by a greater intake of infective larvae from the pasture after the first rains. Genetic correlations among FWEC in the respective seasons were moderate to high, ranging from 0.55 to 0.89. Phenotypic correlations, on the other hand, were significant but lower in magnitude, ranging from 0.09 to 0.16. These results provide useful information for developing strategies for the genetic improvement of ovine resistance to gastrointestinal nematodes under Mediterranean conditions in South Africa by using FWEC as an indicator trait.

## Introduction

The profitability of sheep production relies, in part, on the efficient application of proper strategies to reduce nematode infestations under challenge conditions. At present, the major control strategy for nematodes is the use of chemical drenches to treat and prevent production and economic losses associated with infestation. However, development of parasite resistance to chemicals is common (Rialch, Vatsya & Kumar 2013; Swarnkar & Singh 2011; Waller 1994). In addition, consumer perception is in favour of the usage of minimum chemicals during the meat production process. These developments force sheep producers and researchers to consider alternative strategies relying less on chemicals, like an integrated pest management programme, including selection for parasite resistance to control parasitism over the long term (Khusro et al. 2004).

Genetic variation for ovine resistance to nematode infestation is well documented. Genetic variation exists among different breeds (Baker et al. 1999, 2002; Gruner et al. 2003; Nimbkar et al. 2003) and within breeds (Morris et al. 2000; Woolaston & Windon 2001), when considering different nematode species. The most commonly used criterion in temperate parts to indirectly measure parasite resistance is faecal worm egg count or FWEC (Cloete et al. 2007; Matebesi-Ranthimo et al. 2014; Mpetile et al. 2015). Alternative approaches like applying the FAMACHA^©^ set of measurements (Bath & Van Wyk 2009) may also play a role in helminth control in the summer rainfall areas of South Africa. However, it may not be as directly applicable to dryland conditions in the Mediterranean part of South Africa (Cloete, Mpetile & Dzama 2016), leaving FWEC as the main criterion available for selection against gastrointestinal helminths. Heritability estimates of FWEC reported in literature range from 0 to 0.52 (Greeff, Karlsson & Harris 1995; Lôbo, Vieira & Oliveira 2009; Mpetile et al. 2015). Breeding for a reduced FWEC has been successful in Australia (Greeff, Karlsson & Besier 1999; Greeff, Karlsson & Underwood 2006; Karlsson & Greeff 2006) and New Zealand (Morris et al. 2005). So far, no comparable results on response to selection for a lower FWEC have been published for South African conditions.

Environmental conditions during natural challenge are characterised by large seasonal and across-year variation in temperature and rainfall. These factors markedly affect the prevalence of parasite populations on pastures between months within years and across years (Greeff et al. 1995; O’Connor, Walkden-Brown & Kahn 2006), thus affecting the genetic variation observed in FWEC.

Against this background, a historic dataset of FWEC from four Merino lines maintained at the Tygerhoek research farm was utilised to identify the most appropriate time to test sheep for parasite resistance under natural challenge conditions. The genetic and environmental variation in FWEC in three seasons (autumn, spring and winter) was first assessed in a single-trait repeatability model. Subsequently, FWEC in the three seasons were analysed in a three-trait model. This model was used to determine whether FWEC in different seasons was genetically the same trait and also for deriving genetic and phenotypic correlations among FWEC records obtained in the different seasons.

## Materials and methods

The data used in this study came from four lines of Merino sheep that were selected for an increased clean fleece weight with a limit on fibre diameter (Cloete et al. 1998, 2007; Matebesi-Ranthimo et al. 2014), for a reduction in fibre diameter (Cloete, Olivier & Du Toit 2013; Matebesi-Ranthimo et al. 2014), for an increased reproductive efficiency using the ‘wet and dry’ method (Cloete et al. 2007; Matebesi-Ranthimo et al. 2014), as well as an unselected control line (Cloete et al. 1998, 2007, 2013; Matebesi-Ranthimo et al. 2014). The experimental animals were maintained at the Tygerhoek research farm in the southern Cape of South Africa. Details on these lines and selection of the experimental animals, husbandry practices, climate at the experimental site, sampling procedures and experimental design have been reported in the literature cited. Briefly, the progeny of all lines were maintained as single flocks within birth years, but separated on sex within birth year cohorts throughout the trial. The climate at the experimental site is Mediterranean, with an average annual rainfall of 425 mm, 60% of which is expected during winter. The long-term mean daily temperature at the site ranges from 10.2 °C to 22.4 °C. Animals in the study mostly relied on pastures, namely dryland lucerne (*Medicago sativa*) and medics (*M. truncatula*). Animals also grazed oat (*Avena sativa*) fodder crops during winter and spring as well as wheaten stubble in summer. They occasionally received oat grain as a supplement when grazing stubble lands. Standard drenching and husbandry programmes for the region were followed.

In this study there was no direct selection for parasite resistance in either line (Cloete et al. 2007, 2016). Faecal grab samples were obtained from individual animals after drenching was withheld for a period of at least 10 weeks in autumn, winter and spring. Faecal grab samples were sent to Western Cape Provincial Veterinary Laboratory for analysis, using the McMaster technique with a sensitivity of 100 eggs/gram of wet faeces (Van Schalkwyk et al. 1994). The pathogen species commonly present under dryland conditions in the region were a mixture of *Teladorsagia, Trichostronglyus* and *Nematodirus* spp. (Reinecke 1994), although traces of *Haemonchus contortus* might have been observed (Cloete et al. 2007). The FWEC data were transformed to natural logarithms (after 100 was added to account for zero counts) to reduce the variation in untransformed FWEC and also to normalise the distribution (Table 1).

**TABLE 1:** Descriptive statistics for faecal worm egg counts of Merino sheep before and after transformation.

Trait	Mean ± SD	Range	CV%	Skewness	Kurtosis
FWEC 1	891 ± 1570	0–37 200	177.30	8.81	165.37
FWEC 2	524 ± 1001	0–14 900	191.10	5.17	46.44
FWEC 3	665 ± 920	0–14 300	138.40	3.77	33.32
Log FWEC1	6.17 ± 1.12	4.61–10.53	19.78	0.18	−1.00
Log FWEC2	5.72 ± 1.12	4.61–9.62	19.60	0.61	−0.62
Log FWEC3	6.04 ± 1.12	4.61–9.57	18.62	0.14	−1.17

FWEC1, untransformed faecal worm egg count in autumn; FWEC2, untransformed faecal worm egg count in winter; FWEC3, untransformed faecal worm egg count in spring; log FWEC1, log-transformed faecal worm egg count in autumn; log FWEC2, log-transformed faecal worm egg count in winter; log FWEC3, log-transformed faecal worm egg count in spring.

The data were initially analysed for fixed effects and variance components across seasons using a single-trait repeatability model analyses in ASREML (Gilmour et al. 2009). To obtain the best operational model, the fixed effects of selection line (as reported previously), birth year (1997–2000), birth type (multiple or single), sex (ram or ewe), age of dam (2–7+ years) and season (autumn, winter or spring) were fitted. In addition, all two-factor interactions were initially included in the model but only the birth year x sex interaction and the birth year x season interaction were statistically significant and retained in the operational model along with the other significant fixed effects for subsequent analysis. Random effects were then added to the model, giving the following models for analyses (matrix notation):

y=Xb+e [Eqn 1]

$$y=Xb+Z_{1}a+e$$ [Eqn 2]

$$y=Xb+Z_{1}a+Z_{2}c+e$$ [Eqn 3]

In these analyses, *y* was a vector of observations for log-transformed FWEC across seasons, and b, a and c were vectors of fixed effects, direct genetic variances and animal permanent environmental (PE) variances respectively. X, Z_1_ and Z_2_ represented the corresponding incidence matrices relating the respective effects to y and e was the randomly distributed vector of residuals. It was assumed that:

$$V(a)=A\sigma_{a}^{2};\text{V}(\text{c})=\text{I}\sigma_{\text{c}}^{2};\text{V}(\text{e})=\text{I}\sigma_{\text{e}}^{2}$$ [Eqn 4]

with A representing the numerator relationship matrix; I representing identity matrices; $\sigma_{\text{a}}^{2},\sigma_{\text{c}}^{2}$ and $\sigma_{\text{e}}^{2}$ being the direct genetic, animal PE and residual variances, respectively. Ratios corresponding to additive genetic and PE variances were computed from these estimates and were expressed relative to the total phenotypic variance.

The later analysis assumed equal genetic variances for log-transformed FWEC across seasons, as well as unity genetic correlations among expressions of log-transformed FWEC in each season. To test these assumptions, the analyses were followed by within-season single-trait analyses for records obtained during autumn, winter and spring respectively. To obtain the best operational model for these analyses, the fixed effects of selection line (as reported previously), birth year (1997–2000), birth type (multiple or single), sex (ram or ewe) and age of dam (2–7+ years) were fitted within seasons. In addition, all two-factor interactions were initially included in the model but only the birth year x sex interaction was retained in the operational model along with the other significant fixed effects for subsequent analyses. Random effects were then added to the operational model, resulting in the following models for analyses in matrix notation:

y=Xb+e [Eqn 5]

$$y=Xb+Z_{1}a+e$$ [Eqn 6]

$$y=Xb+Z_{1}a+Z_{2}c+e$$ [Eqn 7]

$$y=Xb+Z_{1}a+Z_{3}m+e$$ [Eqn 8]

[*Co variance* (a, m) = 0]

$$y=Xb+Z_{1}a+Z_{3}m+Z_{2}c+e$$ [Eqn 9]

[*Co variance* (a, m) = *Aσ*_am_]

In these analyses, y was a vector of observations for log-transformed FWEC within seasons, and b, a, m and c were vectors of fixed effects, direct genetic variances, maternal genetic variances and maternal PE variances respectively. X, Z_1_, Z_2_ and Z_3_ represented the corresponding incidence matrices relating the respective effects to y and e was the randomly distributed vector of residuals. A was the numerator relationship matrix and σ_am_ the covariance between direct and maternal additive genetic effects.

It was assumed that:

$$V(a)=A\sigma_{a}^{2};\text{V}(\text{m})=\text{A}\sigma_{\text{m}}^{2};\text{V}(\text{c})=\text{I}\sigma_{\text{c}}^{2};\text{V}(\text{e})=\text{I}\sigma_{\text{e}}^{2}$$ [Eqn 10]

with I representing identity matrices; $\sigma_{\text{a}}^{2},\sigma_{\text{m}}^{2},\sigma_{\text{c}}^{2}$ and $\sigma_{\text{e}}^{2}$ being the direct genetic, maternal genetic, maternal PE and residual variances respectively.

Ratios corresponding to additive genetic and permanent environmental variances were computed from these repeatability and single-trait within-season analyses. These variances were expressed relative to the total phenotypic variance. Likelihood Ratio tests (LRT) were used to test the contribution of each random term to improvements in the operational model for significance. The single-trait within-season analyses were followed by a three-trait analysis treating FWEC in different seasons as separate traits. This analysis also allowed the calculation of season-specific heritability estimates, as well as genetic and phenotypic correlations among FWEC expressions in different seasons. The full pedigree file, including 4650 animals born between 1989 and 2000 as the progeny of 357 sires and 1447 dams, was used in all analyses.

## Results

**FIGURE 1:**
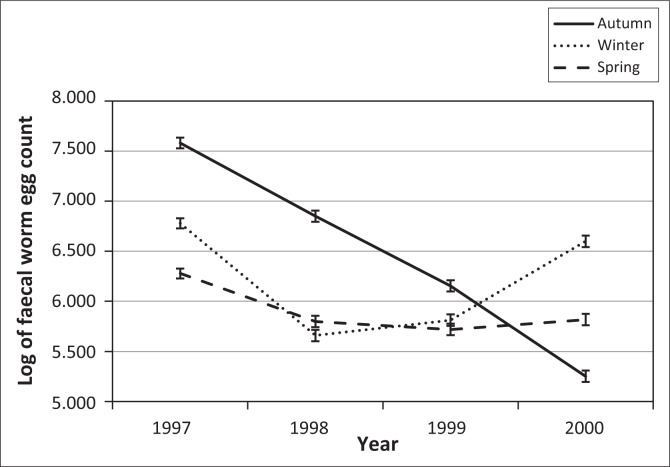
Interaction of year by season for the natural log of faecal worm egg count in the study population (log-transformed least-squares means ± SE).

The descriptive statistics of FWEC showed an extreme variation and a non-normal distribution before transformation, as shown in Table 1. The distribution of the data was improved by transformation to natural logs resulting in coefficients of variation (CV) that were below 20%. Only fixed effect results obtained from the log-transformed analysis on the repeatability model analysis will be presented. The fixed effects of selection line, birth year, sex, season, the sex x birth year interaction and the season x birth year interaction all influenced the data significantly (*p* < 0.001). Sex x birth year interactions are commonly found in similar literature and will not be reported. Seasonal means for FWEC varied quite markedly across years (Figure 1). Log-transformed FWEC means in autumn declined quite markedly from 1997 to 2000; those recorded in winter were higher in 1997 and 2000 and lower in 1998 and 1999, while those recorded in spring declined from 1997 to 1998 to stabilise at the lower levels.

Log-likelihood values for the repeatability model analysis on log-transformed FWEC amounted to -1932.02 for the fixed effect operational model prior to the addition of the additive genetic effect of animal. When animal genetic effects were added to the analysis, the Log-likelihood value improved to -1904.87 (*p* < 0.05 tested as a Chi-square statistic at 1 degree of freedom). The addition of animal permanent environment as another random effect did not result in a further improvement of the Log-likelihood value, which stayed at -1904.87. Log-likelihood values for the subsequent analyses on within-season FWEC accordingly suggested a significant improvement from the model with fixed effects only to the model also including direct additive genetic variation for log-transformed FWEC only in winter and spring (*p* < 0.05; Table 2), but not in autumn (*p* > 0.05). The adding of additional random effects failed to result in further improvements to the goodness of fit of the models fitted in winter and spring.

**TABLE 2:** Log-likelihood ratios for single-trait random effects model fitted to faecal worm egg count data of the Tygerhoek Merino flock with the ‘best’ model in bold.

Model	FWEC1	FWEC2	FWEC3
Fixed	**-500.908**	−556.152	−757.517
Fixed +h^2^	−500.908	**-547.427**	**-750.294**
Fixed +h^2^ + m^2^	−499.296	−547.427	−750.294
Fixed +h^2^ + c^2^	−499.876	−547.070	−750.294
Fixed +h^2^ + m^2^ + c^2^	−499.296	−547.070	−750.294

FWEC1, log-transformed faecal worm egg count in autumn; FWEC2, log-transformed faecal worm egg count in winter; FWEC3, log-transformed faecal worm egg count in spring.

Log-transformed FWEC was lowly heritable in the across-season repeatability model analysis at 0.09 ± 0.02. The derived season-specific heritability estimates of log-transformed FWEC were 0.07 ± 0.05 in autumn, 0.13 ± 0.04 in winter and 0.19 ± 0.05 in spring (Table 3). Relative to the standard errors associated with these heritability estimates, the estimate derived in spring were higher than the one derived in autumn. These results therefore suggested significant genetic variation for FWEC in winter and spring, but not in autumn as was also suggested by the Log-likelihood values in Table 2. Selection for a lower FWEC can thus result in genetic gains when data recorded during winter and spring are used.

**TABLE 3:** Variance components and ratios (±SE) for log-transformed faecal worm egg counts estimated from the three-trait analysis with genetic (above diagonal) and phenotypic correlations (below diagonal) between expressions of faecal worm egg count in different seasons.

Trait analysed	Log FWEC1	Log FWEC2	Log FWEC3
**Variance components**			
$\sigma_{\text{a}}^{2}$	0.046	0.086	0.166
$\sigma_{\text{p}}^{2}$	0.639	0.674	0.880
**(Co)variance ratios (h^2^ on the diagonal in bold)**			
Log FWEC1	**0.07 ± 0.05**	0.89 ± 0.34	0.55 ± 0.30
Log FWEC2	0.12 ± 0.02	**0.13 ± 0.04**	0.78 ± 0.18
Log FWEC3	0.09 ± 0.03	0.16 ± 0.03	**0.19 ± 0.05**

$\sigma_{\text{a}}^{2}$, direct additive variance; $\sigma_{\text{p}}^{2}$, phenotypic variance; h^2^, direct, additive heritability; Log FWEC1, log-transformed faecal worm egg count in autumn; Log FWEC2, log-transformed faecal worm egg count in winter; Log FWEC3, log-transformed faecal worm egg count in spring.

Table 3 also presents genetic and phenotypic correlations among FWEC in different seasons of the year. The genetic correlations among FWEC records from different seasons ranged from moderate to high at 0.55–0.89, suggesting that selection for a reduced FWEC in one season would also improve FWEC in other seasons. Phenotypic correlations, on the other hand, were significant but low and ranged from 0.09 to 0.16.

## Ethical considerations

The animals were maintained, managed and recorded under clearance from the Departmental Ethical Committee for Research on Animals at the Western Cape Department of Agriculture (DECRA reference number R12/76).

## Discussion

Extreme individual variation of FWEC data is commonly reported in literature, ranging from 0 to 50 000 epg (Cloete et al. 2007; Khusro et al. 2004; Matebesi-Ranthimo et al. 2014; Mpetile et al. 2015; Snyman 2007). Log transformations were traditionally applied to reduce the individual variation of FWEC and to improve the properties of the data (Cloete et al. 2007; Matebesi-Ranthimo et al. 2014; Mpetile et al. 2015; Safari & Fogarty 2003). In a study by Safari and Fogarty (2003) 14 of 26 heritability estimates for FWEC were log-transformed, resulting in mean values that ranged from 6.88 to 7.49 after transformation. Cloete et al. (2007), Matebesi-Ranthimo et al. (2014) and Mpetile et al. (2015) reported highly variable, skewed and leptokurtic data for FWEC before transformation. Log transformation also improved the properties of their data, resulting in CV that were below 20%. Similar results were reported in this study. The high CV value obtained in this study indicates sufficient phenotypic variation, which in turn has a potential to allow genetic gains should selection for low FWEC be desired.

The results pertaining to the birth year x sex interaction were consistent with those previously reported (Cloete et al. 2007; Mpetile et al. 2015). Previous research attributed this interaction to a lack of control of the challenge on the pastures utilised by the ram and ewe hoggets, which are grazing in single-sex flocks at that stage. A significant interaction of season with year was also observed in this study, suggesting that there is no uniform environment. It is conceivable that short-term climatic conditions hugely impact on log-transformed FWEC, resulting in these trends (Greeff et al. 1995; Kumba et al. 2003; Pandey, Chitate & Nyanzunda 1993; Pfukenyi et al. 2007). Animals are routinely exposed to different climatic conditions as a result; the parasite population also fluctuates with season between years and also across years (Cloete et al. 2007; Greeff et al. 1995; Matebesi-Ranthimo et al. 2014; Mpetile et al. 2015). The general trend for FWEC in relation to year and season is reported in the literature (Greeff et al. 1995; Kumba et al. 2003; Rahman 1992). In a study by Kumba et al. (2003), the least square means for FWEC were 2140 during the warm, wet season, 430 during cold, dry months and 653 during hot, dry months in goats on communal farms of Namibia. Another study on goats by Rahman (1992) has reported a higher FWEC in the wet season, a moderate FWEC in the hot season and lowest values of FWEC in the cold, dry season. Therefore, when planning a breeding programme for parasite resistance, seasonal variation of FWEC should be considered within and across countries.

The across-season heritability derived for this study from the repeatability model was low at 0.09. However, the three-trait analysis suggested that the magnitude of the genetic variance component for log-transformed FWEC was increased by 87% from 0.046 in autumn to 0.086 in winter. The genetic variance component of log-transformed FWEC in spring was increased by an order of magnitude exceeding 3.5 in comparison to FWEC in autumn. Phenotypic variance components were not affected to the same extent, ranging from 0.64 to 0.88 (Table 3). These results seem to suggest that the expression of genetic variation associated with log-transformed FWEC in different seasons differed quite appreciably. The heritability of the natural logarithm of FWEC accordingly amounted to 0.07 in autumn, but it improved appreciably to 0.13 in winter and further to 0.19 in spring, the latter two estimates reaching a level of double the corresponding standard error. The significant (*p* < 0.05) heritability estimates for FWEC in winter and spring coincided with the growth of pastures after the first rains in the Mediterranean environment and the hatching of parasite eggs during the wet season. The better nutritional conditions during this time of the year seem to have improved the ability of resistant hosts to mount an immune response at the genetic level, which in turn affected the expression of parasite resistance, leading to higher heritability estimates. Studies by Nieuwoudt, Theron and Kruger (2002), Cloete et al. (2007) and Matebesi-Ranthimo et al. (2014) conducted in South Africa from data collected in spring reported moderate to high heritability estimates (0.14–0.24) for FWEC of Merino sheep under natural challenge. These results suggest genetic variation in FWEC and that selection for low FWEC would result in future generation gains. A recent study by Mpetile et al. (2015), using data collected in autumn from the Elsenburg Merino flock under natural challenge conditions in South African conditions, reported lower but still significant heritability estimates of 0.10 for FWEC. The present results, as well as those of the latter authors, suggested that genetic variation for parasite resistance is not well expressed in autumn, despite adequate parasite challenge (as reflected by high values for FWEC). Therefore, should selection for low FWEC be desired, the animals must be assessed sometime after the break of season in winter and in spring under Mediterranean conditions, when temperature and rainfall are favourable for the development, survival and migration of infective larvae onto pastures (O’Connor et al. 2006). Our results also accorded with those obtained by Greeff et al. (1995) when estimating the genetic variation of FWEC in Merino lambs in different seasons under Mediterranean conditions in Western Australia. Relative to standard errors associated with heritability estimates, the estimates derived in their study were low in autumn (*h*^2^ = 0.00–0.03), moderate during winter to late spring (*h*^2^ = 0.21–0.25) and reached a high of 0.51 during mid-winter. Khusro et al. (2004) also reported a moderate heritability estimate for cube root transformed FWEC in yearling Merinos (*h*^2^ = 0.22), while a higher heritability estimate of 0.38 was observed in hoggets. In the latter study, the data were collected from commercial farms with limited information on the season of sampling. However, the current study, along with studies by Greeff et al. (1995), Rahman (1992), Rinaldi et al. (2009) and Kumba et al. (2003) on goats reported a significant effect of season on FWEC, suggesting that seasonal variation in genetic variation for FWEC should also be considered when planning breeding programs for an improved parasite resistance.

Overall, the heritability estimates of FWEC in our study under South African conditions, although moderate, were below the mean value of 0.27 that was derived from 16 literature sources documented in a major review article by Safari, Fogarty and Gilmour (2005). In a study by Greeff and Karlsson (1997), heritability estimates for FWEC were 0.40 for Merino weaners and 0.22 for hoggets under natural challenge. Similar results were reported by Yadav et al. (2006) and Morris et al. (2005), with h^2^ estimates for FWEC of respectively 0.24 in Muzaffarnagari sheep and 0.22 in 22-week-old Perendale sheep. Comparable results were reported by Clarke (2002) in Merino sheep, with heritability estimates ranging from 0.18 to 0.40 for weaners, 0.17–0.34 during the post weaning period and 0.15–0.40 in yearlings. Another study on Merino sheep yielded heritability estimates for FWEC ranging from 0.29 to 0.38 for yearlings and from 0.29 to 0.41 for hoggets depending on the model used (Brown, Swan & Gill 2010). A study in New Zealand reported the heritability of FWEC as 0.37 in naturally challenged Romney sheep (Baker et al. 1991). Hence, it is not surprising that selection for low FWEC has resulted in marked genetic gains in New Zealand and Australia (Greeff et al. 1999; Karlsson & Greeff 2006; Morris et al. 2005; Woolaston & Piper 1996). Similar results can also be expected in South Africa if proper data collection measures, as well as the best sampling period for FWEC, are duly noted and effectively applied.

The lower magnitude of heritability estimates for FWEC obtained in our study could be attributed to a greater variation of FWEC between months within seasons and between seasons within years (Greeff et al. 1995). Additionally, the lower magnitude of our heritability estimates for FWEC could be because of the fact that our study used an accuracy of 100 epg wet faeces compared to a greater accuracy of counts in other studies (Vanimisetti 2003). The latter author reported heritability estimates of 0.42, 0.22 and 0.25 at 3, 4 and 5 weeks, respectively, after artificial challenge with *H. contortus* larvae in sheep. FWEC were counted with a sensitivity of 50 epg wet faeces in this case.

Genetic correlations among log-transformed FWEC in different seasons were moderate to high and positive. Log-transformed FWEC thus seems to be governed by mostly the same genes, irrespective of the season. Although genetic correlations among seasonal log-transformed FWEC measures were not significantly different from each other as indicated by their standard errors, it is notable that the genetic correlation between FWEC in autumn and in spring amounted to only 0.55. The large standard errors accompanying genetic correlations involving FWEC in autumn are possibly because of the smaller additive variance component derived in autumn. Moderate to high positive correlations among FWEC measures in autumn, winter and spring observed in this study seem to suggest that individual free-ranging animals susceptible to nematode infestation in one season are likely to be at risk in other seasons as well. Animals that consume higher volumes of the infected pastures are likely to host greater parasite loads (Zajac 2006). A study by Rinaldi et al. (2009) in goats reported a positive relationship between worm burden and parasite load, suggesting that an increase in worm burden would also increase FWEC. Similar results were obtained for goats by Cringoli et al. (2008), where FWEC and worm burdens were positively correlated with each other. Although the latter studies were conducted on goats, their results could be extrapolated to sheep, as small ruminants share many of the same parasite genera and species. Moreover, a strong relationship between FWEC and parasite load supports the use of FWEC to indirectly measure parasite prevalence as well as the level of infestation (Eysker & Ploeger 2000).

Finally, it is relevant to consider whether FWEC and other traits involving host resistance should form part of formal sheep recording in South Africa. Cloete et al. (2014) suggested that disease resistance traits should be considered for inclusion in this recording scheme, as is the case in Australia (Brown et al. 2010, 2015; Khusro et al. 2004) and New Zealand (Pickering et al. 2012). Cloete et al. (2014) argued that hard-to-measure traits like disease resistance should be targeted for genomic selection procedures in the national sheep flock. Clues as to how to achieve this could be taken from Australia and New Zealand, where such initiatives are already in place (Pickering et al. 2015; Swan et al. 2014).

## Conclusion

Genetic variation for FWEC exists. Selection for lower parasite loads is likely to pay dividends in South Africa, as in overseas countries. However, this study suggested that the best time to challenge sheep for deriving breeding values to assist with selection for parasite resistance is sometime after the break of season in winter and/or spring, when greater numbers of infective larvae after the first rains provide an adequate natural challenge under Mediterranean conditions. This study has an implication for the timing of data collection to allow breeding for parasite resistance under South African Mediterranean conditions.
